# Knockdown of lncRNA MALAT1 Alleviates LPS-Induced Acute Lung Injury via Inhibiting Apoptosis Through the miR-194-5p/FOXP2 Axis

**DOI:** 10.3389/fcell.2020.586869

**Published:** 2020-10-07

**Authors:** Chuan-chuan Nan, Ning Zhang, Kenneth C. P. Cheung, Hua-dong Zhang, Wei Li, Cheng-ying Hong, Huai-sheng Chen, Xue-yan Liu, Nan Li, Lixin Cheng

**Affiliations:** ^1^Department of Critical Care Medicine, Shenzhen People’s Hospital, Second Clinical Medical College of Jinan University, First Affiliated Hospital of Southern University of Science and Technology, Shenzhen, China; ^2^Department of Stomatology Center, Shenzhen People’s Hospital, Second Clinical Medical College of Jinan University, First Affiliated Hospital of Southern University of Science and Technology, Shenzhen, China; ^3^School of Life Sciences, The Chinese University of Hong Kong, Sha Tin, China

**Keywords:** MALAT1, FOXP2, miR-194-5p, apoptosis, acute lung injury

## Abstract

**Purpose:**

We aimed to identify and verify the key genes and lncRNAs associated with acute lung injury (ALI) and explore the pathogenesis of ALI. Research showed that lower expression of the lncRNA metastasis-associated lung carcinoma transcript 1 (MALAT1) alleviates lung injury induced by lipopolysaccharide (LPS). Nevertheless, the mechanisms of MALAT1 on cellular apoptosis remain unclear in LPS-stimulated ALI. We investigated the mechanism of MALAT1 in modulating the apoptosis of LPS-induced human pulmonary alveolar epithelial cells (HPAEpiC).

**Methods:**

Differentially expressed lncRNAs between the ALI samples and normal controls were identified using gene expression profiles. ALI-related genes were determined by the overlap of differentially expressed genes (DEGs), genes correlated with lung, genes correlated with key lncRNAs, and genes sharing significantly high proportions of microRNA targets with MALAT1. Quantitative real-time PCR (qPCR) was applied to detect the expression of MALAT1, microRNA (miR)-194-5p, and forkhead box P2 (FOXP2) mRNA in 1 μg/ml LPS-treated HPAEpiC. MALAT1 knockdown vectors, miR-194-5p inhibitors, and ov-FOXP2 were constructed and used to transfect HPAEpiC. The influence of MALAT1 knockdown on LPS-induced HPAEpiC proliferation and apoptosis via the miR-194-5p/FOXP2 axis was determined using Cell counting kit-8 (CCK-8) assay, flow cytometry, and Western blotting analysis, respectively. The interactions between MALAT1, miR-194-5p, and FOXP2 were verified using dual-luciferase reporter gene assay.

**Results:**

We identified a key lncRNA (MALAT1) and three key genes (EYA1, WNT5A, and FOXP2) that are closely correlated with the pathogenesis of ALI. LPS stimulation promoted MALAT1 expression and apoptosis and also inhibited HPAEpiC viability. MALAT1 knockdown significantly improved viability and suppressed the apoptosis of LPS-stimulated HPAEpiC. Moreover, MALAT1 directly targeted miR-194-5p, a downregulated miRNA in LPS-stimulated HPAEpiC, when FOXP2 was overexpressed. MALAT1 knockdown led to the overexpression of miR-194-5p and restrained FOXP2 expression. Furthermore, inhibition of miR-194-5p exerted a rescue effect on MALAT1 knockdown of FOXP2, whereas the overexpression of FOXP2 reversed the effect of MALAT1 knockdown on viability and apoptosis of LPS-stimulated HPAEpiC.

**Conclusion:**

Our results demonstrated that MALAT1 knockdown alleviated HPAEpiC apoptosis by competitively binding to miR-194-5p and then elevating the inhibitory effect on its target FOXP2. These data provide a novel insight into the role of MALAT1 in the progression of ALI and potential diagnostic and therapeutic strategies for ALI patients.

## Introduction

Sepsis is a common condition that can lead to ICU hospitalization and cause damage to multiple organs, among which the lung is the most vulnerable (Kuebler, 2019; Liu et al., 2020b). Acute respiratory distress syndrome (ARDS) and acute lung injury (ALI) are common complications of sepsis that are often life-threatening (Van Wessem and Leenen, 2018; Forrester et al., 2019). Although great progress has been made for the treatment of ALI, the death rate is still as high as 40% (Villar et al., 2014). The pathogenesis of ALI in sepsis is complicated, including the apoptosis of pulmonary cells. In particular, there is a disruption of epithelial cell function and increased alveolar epithelial cell apoptosis in sepsis-induced ALI (Gong et al., 2017; Kimura et al., 2017). Therefore, it is of great interest to improve our understanding of apoptosis in ALI and to formulate effective preventive and treatment strategies.

Long non-coding RNAs (lncRNAs) have provided a novel understanding of the disease mechanisms involved in ALI and may act as novel treatment agents. The lncRNA, metastasis-associated lung adenocarcinoma transcript 1 (MALAT1), was initially described as a biomarker for metastatic lung cancer (Gutschner et al., 2013). It has reported that lower expression levels of MALAT1 modulated inflammatory responses for the alleviation of lung injury by mediating miR-146a in lipopolysaccharide (LPS)-stimulated ALI (Dai et al., 2018). However, the molecular mechanism of MALAT1 on LPS-induced cellular apoptosis in the context of ALI remains unclear.

Long non-coding RNAs may regulate the biological function of miRNAs by acting as competitive endogenous RNAs (ceRNAs) (Liu et al., 2018; Cheng et al., 2020). Prior findings have indicated that miR-194-5p regulates apoptosis and genes targeted by miR-194-5p are enriched in apoptotic and inflammatory cell pathways, such as MAPK (Wang et al., 2019). The overexpression of miR-194 has been shown to alleviate cellular apoptosis in LPS-induced ALI (Xie et al., 2017). Forkhead box P2 (FOXP2), a transcription factor, is involved in chromatin remodeling, which regulates the transcription of downstream genes (Bruce and Margolis, 2002). Previous analyses have found that FOXP2 is widely present in alveolar epithelial cells, in which it acts as a transcriptional inhibitor that suppresses surfactant alveolar type II epithelial cell secretions to participate in the pathophysiological process of lung injury (Shu et al., 2001, 2007). Together, these findings through the miR-194-5p/FOXP2 pathway in order to suppress apoptosis in ALI.

This study first identified ALI-related lncRNAs and mRNAs using microarray expression data of ALI samples and normal lung tissue samples. Then, we investigated MALAT1 levels in LPS-stimulated human pulmonary alveolar epithelial cells (HPAEpiC) and discerned its function in LPS-stimulated apoptosis using a cell model of ALI.

## Materials and Methods

### Preprocessing of Gene Expression Profile

The gene expression profile GSE18341 was downloaded from the Gene Expression Omnibus (GEO) database. A total of 32 samples were detected using the platform GPL1261, [Mouse430_2] Affymetrix Mouse Genome 430 2.0 Array (Affymetrix Inc., Santa Clara, CA, United States). Eight ALI samples and eight normal samples were selected for expression analysis. The raw data were normalized through MicroArray Suite 5.0 (MAS5.0) using the “affy” package in the R environment (version 3.61) (Hubbell et al., 2002; Cheng et al., 2016a, b). Genes with multiple probes were scored as average expression and genes with multiple symbols were filtered out (Liu et al., 2019), which reduced the number of genes by about 10%, resulting in 20,814 genes for subsequent analysis.

### Identification of ALI-Related Genes

We selected lncRNAs by searching for the keyword “non-coding” on the Affymetrix GPL1261 platform and 12 lncRNAs were detected (Cheng and Leung, 2018b). We set a | fold change (FC)| value > 1.5 and a Benjamini-Hochberg (BH) adjusted *P* value of < 0.05 as the thresholds to screen differentially expressed lncRNAs (DELs), as well as differentially expressed genes (DEGs). Then, we performed a correlation analysis using Pearson correlation coefficient (PCC) larger than 0.5 as the cutoff value to obtain the genes correlated with the ALI-related lncRNAs. Since microRNAs play a major role in transcriptional regulation and gene expression, we downloaded all RNA–RNA interactions from the RNA Interactome Database (RNAinter) (Lin et al., 2020) and calculated the statistical probability of genes sharing a significantly high proportion of microRNAs with key lncRNAs using the hypergeometric distribution. A *P* value of < 0.05 was considered to be statistically significant. Pathogenesis of ALI is due to the injury of both the lung vascular endothelium and alveolar epithelium. Hence, we only concentrated on genes specifically expressed in lung tissue, which was determined by using “lung” as a keyword to search the Gene Ontology (GO) knowledgebase (Cheng and Leung, 2018a; Li et al., 2020; Liu et al., 2020a). Starbase v2.0 algorithm (Li et al., 2014) was employed to explore miRNAs interacting with lncRNAs and mRNAs.

### Cell Culture

Human pulmonary alveolar epithelial cells was bought through the American Type Culture Collection (ATCC) and maintained in Dulbecco’s modified eagle medium (DMEM) (Thermo Fisher Scientific, Inc.), 10% fetal bovine serum (FBS), and 1% penicillin-streptomycin. The cells were grown in a 5% CO_2_ incubator at 37°C. Transfection was conducted using a Lipofectamine^®^2000 system (Thermo Fisher Scientific, Inc.), as per established guidelines. The miR-194-5p inhibitor and small interfering RNAs (siRNAs) that targeted MALAT1 and FOXP2 were produced by Sangon Biotech Co., Ltd. Their sequences were as follows: si-MALAT1 (5′-CAAGCAGACAGC CCGTGCTGCTT-3′), si-negative control (si-NC; 5′-TTCTCCGAACGTGTCA CGTTT-3′), miR-194-5p inhibitor (5′-UCCACAUGGAGU UGCUGUUACA-3′), and negative control (NC) inhibitor (5′-TTCTCCGA ACGTGTCACGTTT-3′). FOXP2 was cloned into plasmid complementary DNA (pcDNA 3.1) (ov-FOXP2). The empty vector (ov-NC) served as negative control. 50 nM si-MALAT1, 50 nM si-NC, 50 nM miR-194-5p inhibitor, 50 nM NC inhibitor, 1 μg/μl ov-FOXP2, and 1 μg/μl ov-NC were added to HPAEpiC (2 × 10^5^ cells/well) for 24 h prior to conducting the experiments. After 48 h, 1 μg/ml LPS (Sigma-Aldrich Chemical Co., Inc.) was supplemented for 24 h. The LPS dose was chosen based on the results of previous studies (Xu et al., 2014).

### RT-qPCR

Total RNAs were isolated using TRIzol^®^ (Thermo Fisher Scientific, Inc.) and RNase-free DNase I (Promega Corporation). The RNA dose and quality were determined using SMA400 UV-VIS (Merinton Instrument, Ltd.). cDNA was synthesized using a PrimerScript 1st Strand cDNA Synthesis kit (Thermo Fisher Scientific, Inc.). RT-qPCR was conducted using a SYBR^®^ Premix Ex Taq^TM^ II kit (Takara Biotechnology Co.). RT-qPCR thermal cycling conditions were as follows: denaturation for 10 min at 95°C, 40 cycles for 15 s at 95°C, and then 60°C for 30 s. GAPDH and U6 were utilized as controls. Relative RNA and miRNA expression were quantified through the 2^–ΔΔ^ Ct technique. Primer sequences were as follows: MALAT1, forward (F) 5′-TGAGGACAACAGGTGAACGA-3′ and reverse (R) 5′-CCCAAGGCCAACATTACATC-3′; miR-194-5p, F’ 5′-CGCGATGCAGTCTCCTGAGT-3′ and R’ 5′- ACCAACCTATCTGTCCGATGCT-3′; FOXP2, F’ 5′-GAAGACAATGGCATTAAACATGGAGG-3′ and R’ 5′-GAATAAAGCTCATGAGATTTACCTGTC-3′; GAPDH, F’ 5′-ACAACTTTGGTATCGTGGAAGG-3′ and R’ 5′-GCCATCACGCCACAGTTTC-3′; U6, F’ 5′-GCCAGCACCATGCTCTTCTA-3′ and R’ 5′-GGTTCCACAGATGCTCAGGTC-3′. All analyses were conducted in triplicate.

### Cell Viability Assay

Cell counting kit-8 (CCK-8) was utilized to determine the viability of both the treated and untreated HPAEpiC. Transfected cells were placed in three replicates at a density of 1 × 10^4^ cells per well in a 96-well plate with 100 μl of medium and incubated for 0, 24, 48, and 72 h after treatment with 1 μg/ml LPS. Then, the cells were incubated for 2 h in 10 μl of CCK-8 solution. Absorbance (optical density, OD) was assessed at 450 nm using an absorbance reader. The experiment was performed in triplicate.

### Apoptosis Assay

Cells were amassed using trypsin and cleaned three times using precooled PBS. An annexin V–propidium iodide (PI) apoptosis assay kit (Biosea Biotechnology Co., Ltd.) was used to determine apoptosis as per established protocols. The cells were placed in 500 μl of a binding buffer (Biosea Biotechnology Co., Ltd.), treated using 5 μl of PI and Annexin V-FITC, maintained for 20 min at 25°C in the dark, and assessed using a BD FACSCalibur flow cytometer (BD Biosciences) along with FlowJo software.

### Western Blotting Analysis

Human pulmonary alveolar epithelial cells were gathered and lysed using the Radio Immunoprecipitation Assay (RIPA) buffer (Thermo Fisher Scientific, Inc.). Protease inhibitor was then added to the cells. Protein levels were evaluated using the bicinchoninic acid protein assay kit (Thermo Fisher Scientific, Inc.). Protein was analyzed on polyacrylamide sodium dodecyl sulfate gel and transferred onto polyvinylidene fluoride film by electrophoresis. Membranes were placed in blocking buffer, incubated using a primary FOXP2 antibody (diluted at 1:1000) (Abcam, Inc.), and then incubated at 25°C for 1 h with a secondary antibody labeled with peroxidase (diluted at 1:5000) (Abcam, Inc.). Glyceraldehyde-3-phosphate dehydrogenase (GAPDH) (Santa Cruz Biotechnology, Inc.) was utilized as internal control. The protein bands were identified using Odyssey 3.0.

### Luciferase Reporter Assay

The wild-type (WT) or mutant (MUT) MALAT1 and WT or MUT FOXP2 containing the target site of miR-194-5p were cloned in pMIR-REPORT Luciferase vectors (Thermo Fisher Scientific, Inc.). HPAEpiC were placed into six-well plates and transfected with the constructs using the Lipofectamine 2000 system (Thermo Fisher Scientific, Inc.) for 48 h. The Dual Luciferase-Reporter 1000 Assay System (Promega Corporation) was utilized to assess luciferase activity. Renilla luciferase activity was used for normalization.

### Statistical Analysis

All data are shown as mean ± standard deviation (SD). Student’s *t* test was used to evaluate the mean difference between two groups, while ANOVA was used for comparisons between two or more groups. BH adjusted *P* value lower than 0.05 refers to statistical significance. All statistical analyses were conducted using R (version 3.6.3) and SPSS (version 19) software.

## Results

**FIGURE 1:**
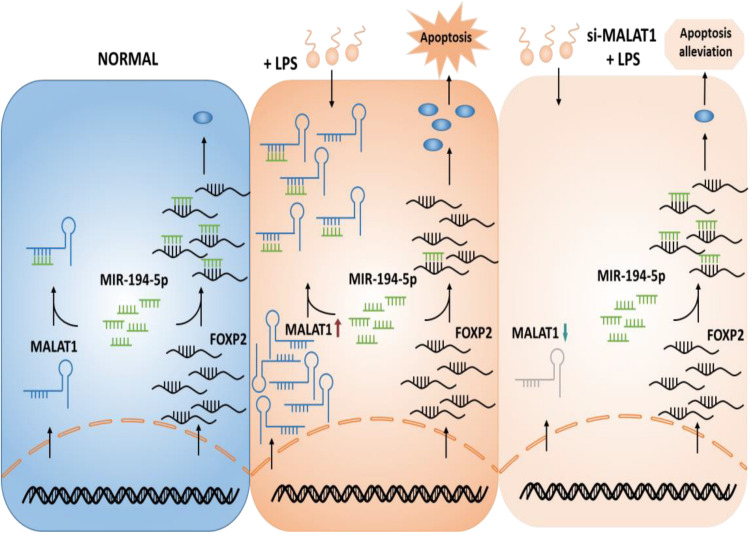
Hypothetical model of the function of MALAT1 in ALI. MALAT1 acts as a sponge of miR-194-5p. Knockdown of MALAT1 alleviates apoptosis of LPS-stimulated HPAEpiC by restoring FOXP2 expression. ALI, acute lung injury; LPS, lipopolysaccharide.

We hypothesized a ceRNA model in ALI that MALAT1 functions as a ceRNA by sponging miR-194-5p to regulate downstream FOXP2 expression and as a result suppresses apoptosis (Figure 1). We aimed to demonstrate that the knockdown of lncRNA MALAT1 alleviates LPS-induced ALI via inhibiting apoptosis by acting as a miR-194-5p sponge to downregulate FOXP2. Transcriptome analysis was carried out and MALAT1 and FOXP2 were identified as ALI regulators. To confirm the suppressing effect of MALAT1 knockdown on LPS-induced HPAEpiC proliferation and apoptosis, CCK-8 assay, flow cytometry, and Western blotting analysis were subsequently performed (see “Materials and Methods”).

### Identification of Malat1 and Foxp2 as ALI Regulators

**FIGURE 2:**
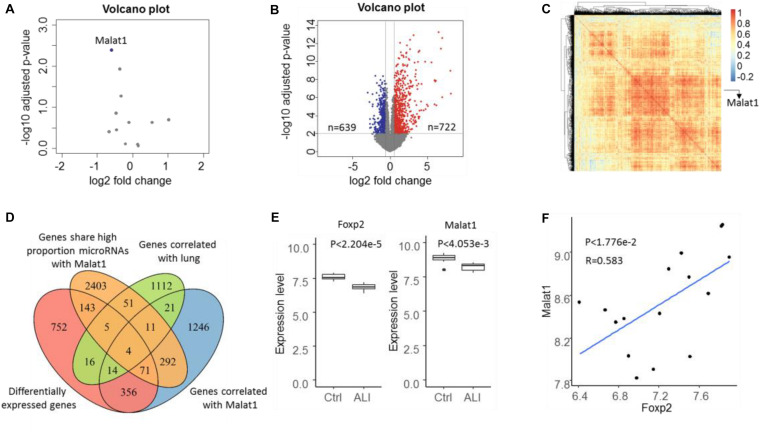
Volcano plot of lncRNA **(A)** and mRNA **(B)** expressions in the microarray dataset GSE18341. Genes with *P* value < 0.01 and | log FC| > 1.5 were considered to be differentially expressed. Blue represents downregulated and red represents upregulated. **(C)** The heatmap depicts the correlation of 2015 genes correlated with Malat1 and most correlations were positive. **(D)** Venn diagram showing the four ALI-related genes determined through the intersection of differentially expressed genes (DEGs), genes correlated with lung, genes correlated with DELs, and genes sharing a significantly high proportion of miRNA targets with Malat1. **(E)** Boxplot illustrating Foxp2 and Malat1 expression levels in control and ALI samples. **(F)** Scatter plot indicating the positive correlation between Foxp2 and Malat1.

Based on the expression profile GSE18341 of mice, 12 lncRNAs were reannotated and only one lncRNA, Malat1, was found to be differentially expressed (Figure 2A). Compared with the control samples, 722 upregulated and 639 downregulated genes were differentially expressed (Figure 2B). Meanwhile, 2015 genes were highly correlated with Malat1 across all the samples with PCC over 0.5 (Figure 2C). By searching for all RNA–RNA interactions, we observed that 2980 genes shared a high proportion of microRNAs with Malat1. To determine lung-specific genes, we screened the Gene Ontology and identified 1234 genes associated with lung tissue. Eventually, we identified three differentially expressed lung-specific genes sharing substantial miRNA targets with Malat1, namely, Eya1, Wnt5a, and Foxp2 (Figure 2D). It has been reported that proteins encoded by Foxp2 are key regulators of apoptosis in the lung (Ren et al., 2020). Importantly, both Malat1 and Foxp2 are differentially expressed and significantly positively correlated (PCC = 0.583, *P* < 1.776e−2), prompting us to investigate the regulatory mechanisms of Malat1 and Foxp2 in ALI (Figures 2E,F).

### LPS Increases MALAT1 Expression and Suppresses HPAEpiC Viability

**FIGURE 3:**
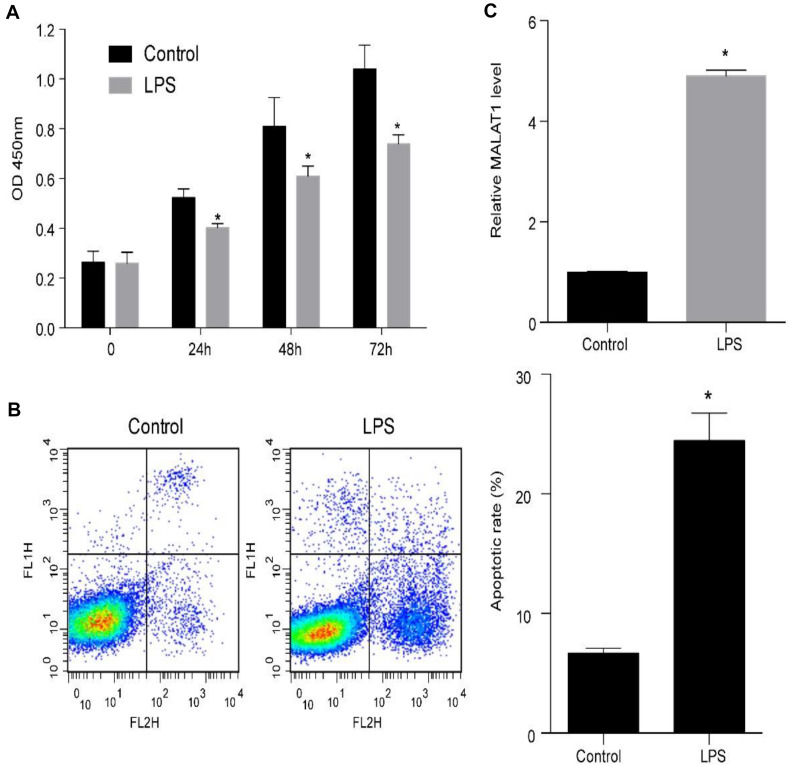
LPS promotes MALAT1 expression and apoptosis, and suppresses the viability of HPAEpiC. **(A)** Viability of HPAEpiC treated with 1 μg/ml LPS analyzed at 24, 48, and 72 h. **(B)** Flow cytometry was used to detect apoptosis of HPAEpiC treated with LPS at 24 h. **(C)** MALAT1 expression in 1 μg/ml LPS-treated HPAEpiC after 24 h was determined using RT-qPCR. ^∗^*P* < 0.05 vs. respective control. OD, optical density; FL1H, fluorescence of PI; FL2H, fluorescence of Annexin V-FITC.

Human pulmonary alveolar epithelial cells were cultured with 1 μg/ml LPS to examine the mechanism of ALI. LPS treatment of HPAEpiC significantly suppressed proliferation and promoted apoptosis (Figures 3A,B). Specifically, the viability (OD 450 nm) of HPAEpiC treated with 1 μg/ml LPS was quantified at 24, 48, and 72 h, respectively, which were significantly decreased in comparison to the untreated counterparts (Figure 3A). Flow cytometry was used to detect the level of apoptosis in treated HPAEpiC at 24 h. The apoptotic rate significantly increased from around 7 to 24% (Figure 3B). Moreover, treatment with LPS substantially promoted an almost fivefold increase in MALAT1 expression (from 0.98 to 4.87, Figure 3C).

### MALAT1 Knockdown Rescues the LPS-Stimulated Effect on Viability and Apoptosis of HPAEpiC

**FIGURE 4:**
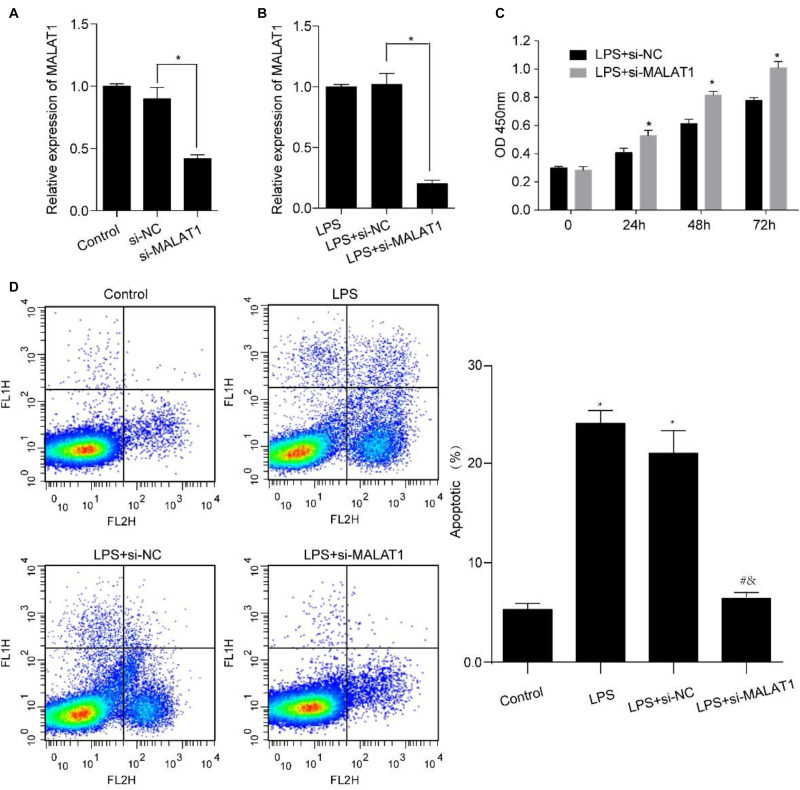
MALAT1 knockdown reverses the LPS-induced effects on viability and apoptosis of HPAEpiC. HPAEpiC transfected with si-MALAT1 or si-NC at 48 h were treated with 1 μg/ml LPS for 24 h. **(A)** RT-qPCR analysis of MALAT1 expression in HPAEpiC after transfection with si-NC or si-MALAT1 at 24 h. **(B)** RT-qPCR analysis of MALAT1 expression in 1 μg/ml LPS-treated HPAEpiC after transfection with si-NC or si-MALAT1 at 24 h. **(C)** Viability was evaluated in HPAEpiC treated with 1 μg/ml LPS and transfected with si-NC or si-MALAT1 at 24, 48, and 72 h. **(D)** Flow cytometry was used to detect the apoptosis of HPAEpiC treated with 1 μg/ml LPS and transfected with si-NC or si-MALAT1. ^∗^*P* < 0.05 vs. respective control; ^#^*P* < 0.05 vs. LPS; ^&^*P* < 0.05 vs. LPS+si-NC.

Human pulmonary alveolar epithelial cells were transfected using si-MALAT1 for 48 h and incubated using 1 μg/ml LPS for 24 h. MALAT1 expression levels decreased significantly in HPAEpiC transfected with si-MALAT1 in comparison to controls, with or without 1 μg/ml LPS stimulation (Figures 4A,B). We observed that MALAT1 knockdown promoted the growth of LPS-treated HPAEpiC. Specifically, the optical density (OD) values of si-MALAT1 were consistently higher than that of si-NC at 24, 48, and 72 h (Figure 4C). Moreover, MALAT1 knockdown inhibited the apoptosis of LPS-stimulated HPAEpiC (Figure 4D). The apoptotic ratios dropped down from over 20% to just about 6% after MALAT1 knockdown. Therefore, MALAT1 knockdown rescued effects caused by LPS treatment.

### MALAT1 Targets miR-194-5p to Mediate FOXP2 Expression

**FIGURE 5:**
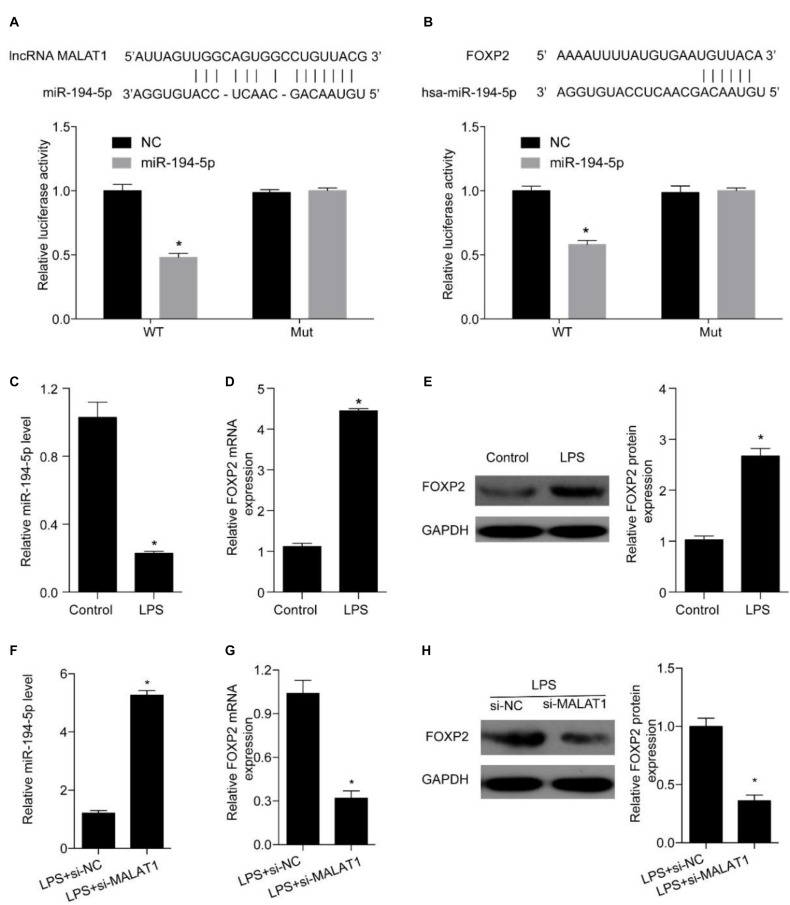
MALAT1 targets miR-194-5p to regulate FOXP2 expression in LPS-treated HPAEpiC. **(A)** The predicted binding sites between MALAT1 and miR-194-5p, with the dual-luciferase reporter gene assay used to verify the relationship between MALAT1 and miR-194-5p. **(B)** The binding sites between miR-194-5p and FOXP2 predicted using dual-luciferase reporter gene assay were used to verify the relationship between miR-194-5p and FOXP2. **(C)** RT-qPCR was used to detect the expression of miR-194-5p in HPAEpiC treated with LPS at 24 h. **(D)** mRNA and **(E)** protein levels of FOXP2 in 1 μg/ml LPS-treated HPAEpiC were evaluated using RT-qPCR and Western blotting analysis, respectively, at 48 h. **(F)** RT-qPCR was used to detect the expression of miR-194-5p in LPS-treated HPAEpiC transfected with si-MALAT1 at 24 h. FOXP2 **(G)** mRNA and **(H)** protein levels in 1 μg/ml LPS-treated HPAEpiC transfected with si-MALAT1 were evaluated using RT-qPCR and Western blotting analysis, respectively, at 24 h. *P < 0.05 vs. respective control.

Next, we explored miRNAs that simultaneously interact with MALAT1 and FOXP2 and miR-194-5p was identified as a candidate (Figures 5A,B). A dual-luciferase reporter assay was conducted to evaluate whether miR-194-5p targeted MALAT1. High miR-194-5p levels substantially lowered luciferase activity associated with WT-MALAT1. However, high miR-194-5p levels barely had an effect on MUT-MALAT1 luciferase activity (Figure 5A). Results of the dual-luciferase reporter assay also suggested that high miR-194-5p levels decreased the luciferase activity associated with WT-FOXP2, and high miR-194-5p levels had no effect on the luciferase activity of MUT-FOXP2 (Figure 5A). Additionally, treatment with 1 μg/ml LPS for 24 h substantially suppressed miR-194-5p levels (Figure 5C) and enhanced FOXP2 levels (Figures 5D,E). MALAT1 knockdown substantially increased the expression levels of miR-194-5p (Figure 5F) and suppressed that of FOXP2 (Figures 5G,H) after 24-h treatment with LPS. These results indicate that MALAT1 knockdown rescues the LPS-stimulated effects exerted on miR-194-5p and FOXP2 levels in HPAEpiC.

### miR-194-5p Inhibitor Reverses the Influence of MALAT1 Knockdown on FOXP2

**FIGURE 6:**
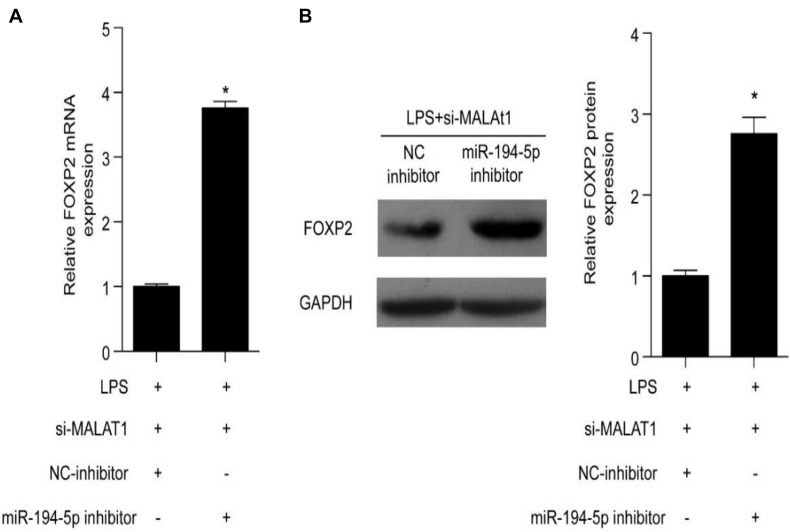
miR-194-5p inhibitor reverses the effect of MALAT1 knockdown on FOXP2 expression in LPS-induced HPAEpiC. **(A)** mRNA and **(B)** protein levels of FOXP2 in 1 μg/ml LPS-treated cells co-transfected with miR-194-5p inhibitor and si-MALAT1 were evaluated using RT-qPCR and Western blotting analysis, respectively, at 24 h. ^∗^*P* < 0.05 vs. respective control.

Forkhead box P2 is a downstream target of MALAT1 and miR-194-5p. A specific inhibitor was utilized to inhibit miR-194-5p levels in order to determine the effect of MALAT1 on LPS-stimulated HPAEpiC. RT-qPCR results suggest that both mRNA and protein levels of FOXP2 were elevated by the miR-194-5p inhibitor (Figures 6A,B). Thus, miR-194-5p inhibition reverses the effect of si-MALAT1 on FOXP2 expression in LPS-treated HPAEpiC.

### FOXP2 Overexpression Reverses the Influence of MALAT1 Knockdown on Viability and Apoptosis

**FIGURE 7:**
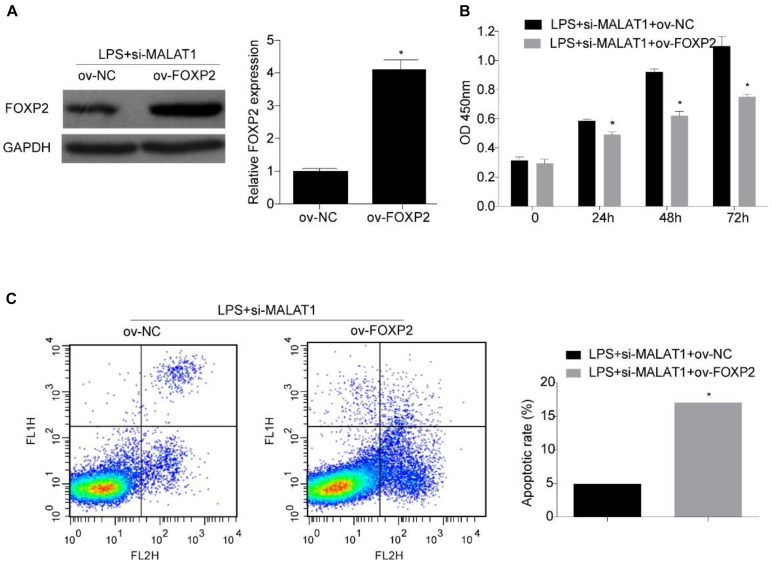
FOXP2 overexpression reverses the effect of MALAT1 knockdown on viability and apoptosis of LPS-treated HPAEpiC. **(A)** FOXP2 expression in 1 μg/ml LPS-treated HPAEpiC co-transfected with ov-FOXP2 and si-MALAT1 was evaluated using Western blotting at 24 h. **(B)** Viability of the HPAEpiC treated with 1 μg/ml LPS and co-transfected with ov-FOXP2 and si-MALAT1 was determined at 24, 48, and 72 h. **(C)** Apoptosis of HPAEpiC treated with 1 μg/ml LPS and co-transfected with ov-FOXP2 and si-MALAT1 was determined using flow cytometry at 24 h. *P < 0.05 vs. respective control.

Next, we investigated whether FOXP2 is required for the suppression effect exerted by the MALAT1–miR-194-5p axis on LPS-induced HPAEpiC. HPAEpiC were co-transfected with ov-FOXP2 and si-MALAT1 for 48 h and followed by treatment with 1 μg/ml LPS for 24 h to assess the effect of FOXP2 on MALAT1. The result of Western blotting analysis indicated that FOXP2 was substantially improved in HPAEpiC co-transfected with ov-FOXP2 and si-MALAT1, in comparison to those co-transfected with ov-NC and si-MALAT1, in LPS-stimulated HPAEpiC (Figure 7A). Moreover, overexpression of FOXP2 suppressed cell viability (Figure 7B) and increased apoptosis (Figure 7C), thereby rescuing the effect exerted by si-MALAT1 on LPS-induced HPAEpiC.

## Discussion

In this study, we developed a LPS-stimulated cell model of ALI *in vitro* and evaluated the function of MALAT1 on apoptosis in LPS-stimulated ALI. The results indicated that MALAT1 knockdown alleviates the apoptosis of LPS-stimulated HPAEpiC via the miR-194-5p/FOXP2 axis (Figure 1). Our results elucidated the regulatory effect directly exerted by MALAT1 on miR-194-5p and FOXP2 levels and mediation of the apoptosis of LPS-treated HPAEpiC.

During the pathophysiological process involved in sepsis-induced ALI, the release of inflammatory factors and mediators causes the death of a large number of structural lung cells, especially alveolar epithelial cells (Han and Mallampalli, 2015). Alveolar epithelial cells show the characteristics of cell apoptosis, such as chromatin condensation, DNA breaks, and overexpression of Bcl-2 protein, in patients with ARDS (Guinee et al., 1997). Apoptosis-related pathways were significantly more active in patients with ARDS (Albertine et al., 2002), and in animal experiments, apoptotic protease inhibitors were found to have reduced the mortality of ALI mice (Kawasaki et al., 2000). Hence, alveolar epithelial cell apoptosis is vital for the pathophysiological process of ALI/ARDS.

Metastasis-associated lung adenocarcinoma transcript 1 plays a crucial role in tumors, angiogenesis, embryonic development, and other pathophysiologies (Lei et al., 2018). Additionally, MALAT1 is closely associated with cell proliferation and apoptosis, and the overexpression of MALAT1 promotes cell apoptosis and inhibits cell proliferation through a variety of regulatory mechanisms (Zhang et al., 2017; Chen et al., 2018). Previous studies have found that MALAT1 upregulation can inhibit cell proliferation in acute kidney injury and neonatal respiratory distress syndrome, promote cell apoptosis, and proinflammatory cytokine expression (Ding et al., 2018; Juan et al., 2018). Nevertheless, low expression of MALAT1 reduced lung injury induced by LPS through the p38 MAPK/p65 NF-κB pathway (Li et al., 2018; Lin et al., 2019). Our study determined that LPS stimulation markedly increased MALAT1 expression levels, inhibited growth, and encouraged the apoptosis of HPAEpiC. Furthermore, MALAT1 knockdown encouraged cell growth and inhibited apoptosis in LPS-stimulated HPAEpiC.

Interference in protein interactions and new undesired protein interactions can cause diseases (Cheng et al., 2017, 2018, 2019), so does the dysfunction of regulatory interactions between miRNAs and genes. miR-194-5p has been reported to regulate cell growth and apoptosis and participate in the pathophysiological processes involved in the development of lung injury (Wang et al., 2017), and it was recognized as a potential target for MALAT1 in this study. Luciferase reporter assay confirmed the direct relationship between MALAT1 and miR-194-5p, suggesting that miR-194-5p is directly targeted by MALAT1. Furthermore, we determined that FOXP2 is a downstream target of miR-194-5p. FOXP2 is widely expressed in alveolar epithelial cells, in which it acts as a transcriptional inhibitor and participates in pathophysiological processes of lung injury (Shu et al., 2001, 2007). Also, FOXP2 regulates proliferation and apoptosis in a variety of malignant tumors. For instance, miRNAs were reported to control the growth and apoptosis of gastric cancer cells by acting on FOXP2 (Jia et al., 2016). The loss of FOXP2 expression in pancreatic tumors leads to an accelerated growth of pancreatic tumor cells and increased blood metastasis (Diao et al., 2018).

In this study, luciferase reporter assay was used to validate the direct regulation between FOXP2 and miR-194-5p, suggesting that FOXP2 is directly targeted by miR-194-5p. Moreover, miR-194-5p expression levels decreased and FOXP2 levels increased in LPS-stimulated HPAEpiC, while MALAT1 knockdown enhanced miR-194-5p levels and suppressed FOXP2 levels. This result indicated that MALAT1 knockdown rescued LPS-stimulated effects on miR-194-5p expression and FOXP2 in HPAEpiC. Further research indicated that miR-194-5p inhibition reversed the effect of si-MALAT1 on FOXP2 levels in LPS-stimulated HPAEpiC and FOXP2 overexpression significantly inhibited cell viability and promoted apoptosis, overturning the influence of si-MALAT1 on LPS-stimulated HPAEpiC. Therefore, MALAT1 knockdown encouraged cell viability and suppressed apoptosis by upregulating miR-194-5p and downregulating FOXP2 in LPS-treated HPAEpiC.

## Conclusion

Our findings reveal that MALAT1 functions as a ceRNA to mediate LPS-stimulated apoptosis of HPAEpiC via sponging miR-194-5p and stimulating FOXP2 expression. Further research is warranted to explore the function of MALAT1 and to confirm the effect of MALAT1 inhibition on disease mechanisms that lead to the development of ALI.

## Data Availability Statement

The original contributions presented in the study are included in the article/Supplementary Material, further inquiries can be directed to the corresponding authors.

## Author Contributions

LC and CN conceived the idea and drafted the manuscript. NZ performed the data analysis. CN and NL performed the bench experiments. LC and XL supervised this project, while HZ, WL, CH, and HC helped interpret the results and provided suggestions for improvement. All authors contributed to the article and approved the submitted version.

## Conflict of Interest

The authors declare that the research was conducted in the absence of any commercial or financial relationships that could be construed as a potential conflict of interest.
