# Supplementary figures and images for: Knockdown of lncRNA MALAT1 Alleviates LPS-Induced Acute Lung Injury via Inhibiting Apoptosis Through the miR-194-5p/FOXP2 Axis
Source: Front Cell Dev Biol. 2020 Oct 7;8:586869. doi: 10.3389/fcell.2020.586869 (PMC7575725; doi:10.3389/fcell.2020.586869)

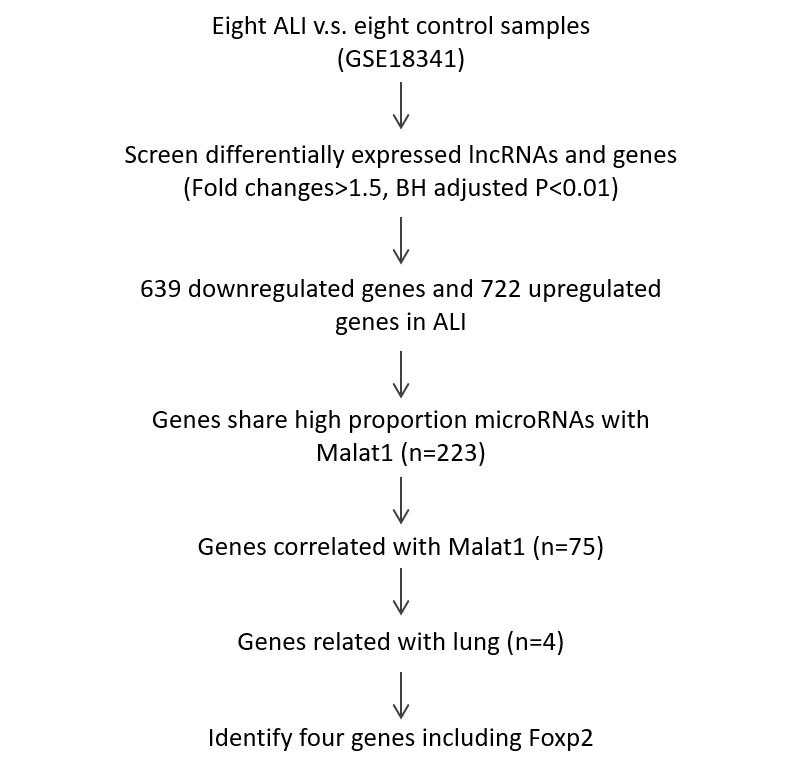


**Figure S1**. Diagram showing the procedure of filtering out Foxp2 from the expression profile.

Supplement: Supplementary file 1 [file Table_1.DOCX]
